# The processing of coherent global form and motion patterns without visual awareness

**DOI:** 10.3389/fpsyg.2014.00195

**Published:** 2014-03-14

**Authors:** Charles Y. L. Chung, Sieu K. Khuu

**Affiliations:** The School of Optometry and Vision Science, The University of New South Wales, KensingtonNSW, Australia

**Keywords:** motion perception, speed discrimination, perceived speed, continuous flash suppression, conscious awareness

## Abstract

In the present study we addressed whether the processing of global form and motion was dependent on visual awareness. Continuous flash suppression (CFS) was used to suppress from awareness global dot motion (GDM) and Glass pattern stimuli. We quantified the minimum time taken for both pattern types to break suppression with the signal coherence of the pattern (0, 25, 50, and 100% signal) and the type of global structure (rotational, and radial) as independent variables. For both form and motion patterns increasing signal coherence decreased the time required to break suppression. This was the same for both rotational and radial global patterns. However, GDM patterns broke suppression faster than Glass patterns. In a supplementary experiment, we confirmed that this difference in break times is not because of the temporal nature of GDM patterns in attracting attention. In Experiment 2, we examined whether the processing of dynamic Glass patterns were similarly dependent on visual awareness. The processing of dynamic Glass patterns is involves both motion and form systems, and we questioned whether the interaction of these two systems was dependent on visual awareness. The suppression of dynamic Glass patterns was also dependent on signal coherence and the time course of suppression break resembled the detection of global motion and not global form. In Experiment 3 we ruled out the possibility that faster suppression break times was because the visual system is more sensitive to highly coherent form and motion patterns. Here contrast changing GDM and Glass patterns were superimposed on the dynamic CFS mask, and the minimum time required for them to be detected was measured. We showed that there was no difference in detection times for patterns of 0 and 100% coherence. The advantage of highly coherent global motion and form patterns in breaking suppression indicated that the processing and interaction of global motion and form systems occur without visual awareness.

## INTRODUCTION

It has been well established that the visual system analyses information in the scene in at least two computationally distinct steps in which *local* scene statistics (representing basic visual features such as orientation, contrast, and color) are first extracted before being integrated to detect *global* image properties such as the overall shape and motion of objects (e.g., Marr, 1983). Global image properties are most informative in determining the spatial layout and in the recognition of objects, which is most critical for visually guided behavior.

The processing of visual information at different levels of image description is perhaps most representative in the manner in which form (i.e., the shape and structure of objects) and motion (i.e., the speed and direction of objects) information is extracted by the visual system (e.g., Glass, 1969; Newsome and Pare, 1988; Edwards and Badcock, 1994; Khuu and Badcock, 2002; Khuu et al., 2011). It has been well established that the detection and analysis of motion occurs in at least two computational steps (see Nishida, 2011 for a review). Initially, the visual system derives local estimates of motion, before these estimates are integrated at a later stage to provide an indication of the overall or global motion of objects (Adelson and Movshon, 1982; Williams and Sekuler, 1984; Wilson, 1994). It is believed that this two-stage analysis of motion is sub-served by neural areas located along the dorsal visual pathway projecting from primary visual cortex (V1) to the parietal cortex (e.g., Ungerleider and Mishkin, 1982; Livingstone and Hubel, 1987; Goodale and Milner, 1992; Van Essen et al., 1992). In particular, neurons in V1 are restricted in their spatial extent of analysis, and only obtain an estimate of local motion, before outputting to higher cortical areas located along the dorsal pathway, such as middle temporal (MT) and medial superior temporal (MST; e.g., Adelson and Movshon, 1982; Newsome and Pare, 1988; Duffy and Wurtz, 1991a,b; Orban et al., 1995). Cells in these areas have large receptive fields and function by pooling the responses of local motion detectors to derive an estimate of the global or overall direction and speed of objects. In comparison the analysis of form information is sub-served by neurons in visual areas located along a ventral projection from V1 to the inferior temporal cortex (Ungerleider and Mishkin, 1982; Livingstone and Hubel, 1987; Goodale and Milner, 1992). In particular local form information, such as orientation, is extracted by orientation-tuned cells in V1, before their outputs are combined in higher cortical areas such as V4 to detect complex forms such as global concentric and radial structures (Gallant et al., 1996; Wilson and Wilkinson, 1998; Wilkinson et al., 2000; Smith et al., 2002).

While understanding the functioning of neural mechanisms responsible for the computation of motion and form continues to be the focus of much research, in recent years attention has been turned toward understanding the factors that might influence their processing. In particular, a question that has recently received much interest is whether visual awareness (i.e., explicit conscious perception) is a necessary requirement for detection and processing of global form and motion information (e.g., Kim and Blake, 2005; Lin and He, 2009). Previous studies have established that under appropriate conditions, local motion information that is suppressed from awareness to the visual system continues to be processed such that it influences subsequent visual discrimination and or judgements (e.g., Tsuchiya and Koch, 2005; Tong et al., 2006; Tsuchiya et al., 2006; Maruya et al., 2008). For example, a number of behavioral studies using binocular rivalry have demonstrated that binocular suppression of an adapting stimulus does not eliminate its ability to generate a motion after effect (MAE) in a subsequently presented test static stimulus (e.g., see Lehmkuhle and Fox, 1975; Blake, 1977; O’Shea and Crassini, 1981). Additionally, the processing of global motion has also been shown to occur without visual awareness. Kaunitz et al. (2011) measured the MAE produced by adaptation to a complex (spiral) motion stimulus suppressed from awareness using the more powerful method of continuous flash suppression (CFS, see Tsuchiya and Koch, 2005) to achieve prolonged and stable visual suppression. Here a flickering mask (e.g., a Mondrian pattern changing at approximately 10 Hz) is presented to one eye, and the to-be-suppressed target image to the other. Under CFS, the observer perceives the flickering mask, and not the adapting stimulus without perceptual alternation and for extended durations sufficient to generate an MAE. Using CFS, Kaunitz et al. (2011) demonstrated that, despite the adapting complex motion stimulus being suppressed from awareness, it was effective in generating a MAE, though the extent of the MAE was attenuated relative to visible conditions. This suggests that the integration of local motion information occurs without visual awareness. This finding is further corroborated by recent findings that highly coherent global dot motion (GDM) patterns, which probes the processing global motion (see Williams and Sekuler, 1984; Newsome and Pare, 1988), break CFS more often than patterns with no global motion coherence (see Kaunitz et al., 2013). This selectivity to highly coherent patterns provides corroborative evidence which further indicates that the visual system is able to integrate local motion signals without awareness. In summary, these findings show that the processing of motion information and perceptual change can occur largely without the need for the visual awareness, and conscious vision and attention might serve to enhance the processing of motion. For example, previous studies have demonstrated that a stronger MAE is produced with visible stimuli compared to those that were suppressed from awareness using CFS (see Maruya et al., 2008; Kaunitz et al., 2013). This suggests an advantage for visible motion in activating motion detectors. Indeed, attention is known to increase the response of neurons of motion selective mechanisms in MT (e.g., Treue and Maunsell, 1996; Seidemann and Newsome, 1999).

While previous research has provided evidence for the unconscious processing of local and global motion, it remains largely unclear what role visual awareness plays in the integration and processing of global form information. While basic stimulus features such as the color, orientation and luminance of objects has been shown to be processed without awareness (e.g., Blake and Fox, 1974; White et al., 1978; He and MacLeod, 2001; Harris et al., 2011), whether conscious vision necessitates global form processing remains unresolved. Indeed previous studies have shown that the detection of the form of complex objects such as faces is driven by visual awareness and cannot be processed without them being visible to the observer. For example, adaptation to faces is dependent on awareness of the adapting stimulus (see Moradi et al., 2005; Stein and Sterzer, 2011). Though, this finding is not entirely clear as certain aspects of face processing such as facial expression can be processed without visual awareness (see Jiang and He, 2006; Adams et al., 2010, though see Yang et al., 2010). Additionally Harris et al. (2011) demonstrated that visual awareness of tokens (suppressed using CFS) that generate a Kanizsa shape is needed for the processing of illusory contours. This suggests that perceptual grouping and integration of form might be largely driven by visual awareness and is a key characteristic of conscious vision. However, recent behavioral evidence has challenged this notion. For example, using Kanizsa figures that were completely suppressed from awareness using CFS, Wang et al. (2012) demonstrated that patterns with tokens appropriately placed to produce illusory shapes broke suppression (and became visible to the observer) faster and more often than when tokens were randomly configured and thus no global illusory shape was evident. Contrary to the assertions of Harris et al. (2011), Wang et al. (2012) argued that this configuration advantage is evidence that the visual system perceptually groups local features without awareness to generate illusory form. In agreement Mudrik et al. (2011) showed that the visual system is able to integrate information to determine the spatial layout of the visual scene without awareness. In particular, objects on incongruent backgrounds broke suppression faster than when the background was congruent. Given these differences in findings, it remains unclear the degree to which visual awareness modulates the processing of global form information. Perhaps a limitation is that these previous studies have not used stimuli that most directly reflect the integration of local form information. Additionally, note that the *recognition* of stimuli such as faces, illusory contours and object-background configurations might require additional top-down processing involving feedback projections from higher cortical areas (e.g., Blake, 1977; Lin and He, 2009), and it might be that this process is dependent (or not dependent) on awareness and not the integration of form information per se.

**FIGURE 1:**
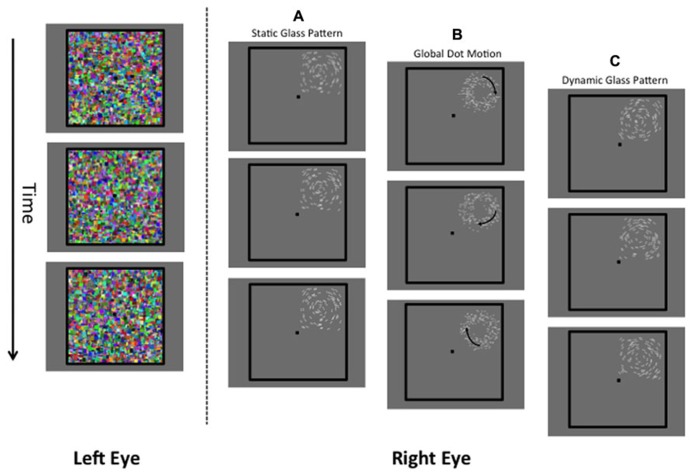
**A schematic of the stimuli used in the present study.** The left eye is presented with a dynamic Mondrian mask (changing randomly at 10 Hz). The right eye is presented with the target stimulus, which was a static Glass pattern **(A)**, global dot motion stimulus **(B)**, or a Dynamic Glass pattern **(C)**.

In the present study we sought to contribute to understanding by investigating whether visual awareness modulates the processing of global form in Glass patterns. Glass patterns (after Glass, 1969) are random dot stimuli consisting of dot-pairs with the same polarity (dipoles) configured to convey global structure (e.g., concentric structure in **Figure 1B**). Glass patterns are particularly useful because their analysis by the visual system is well understood (e.g., Gallant et al., 1996; Bair et al., 2002), and reflects both local and global levels of computation: the orientation of local dipoles is initially extracted, and then combined at a later stage where the global form of the pattern can be determined (e.g., Wilson and Wilkinson, 1998; MacGraw et al., 2004; Wilson et al., 2004; Khuu and Hayes, 2005; Khuu et al., 2011). Thus, Glass pattern stimuli are ideal for investigating the processing of global form as they provide a direct probe of the mechanisms responsible for form integration that is not driven by top down influences unlike previously used stimuli (mentioned above) such as illusory contours and faces. Using the CFS break technique of Jiang et al. (2007), we suppressed Glass patterns from awareness and determined the time taken for them to break suppression and to be consciously perceptible to observers. Here, unlike standard CFS procedures in which the target stimulus can be suppressed for long periods, in CFS break the contrast of the stimulus is systematically increased to force it to break suppression. The “break time” required for this to occur provides an independent measure of the degree to which the pattern is processed unconsciously. In particular as indicated by Jiang et al. (2007) faster break times (relative to a null condition) to a stimulus suppressed from awareness might be indicative of unconscious processing of that stimulus.

It has been demonstrated that the structural coherence of the pattern modulates the degree to which a global motion breaks suppression with highly coherent patterns breaking suppression more often than those consisting of randomly moving dots (see Kaunitz et al., 2013). We adopted this procedure in Experiment 1 and systematically modulated the signal coherence (the proportion of dipoles contributing to the global form of the stimulus) of Glass patterns under CFS suppression. Additionally, for comparison, we also examined the suppression of GDM patterns under the same stimulus conditions. As noted by Kaunitz et al. (2013), global motion processing occurs without visual awareness and this allows for direct comparison with the processing of form. In Experiment 1 we questioned whether the time taken for Glass patterns to break CFS suppression shows an advantage for highly coherent patterns as they do for GDM patterns. In Experiment 2 we continue this examination by determining whether signal coherence influences the break times with dynamic Glass patterns. Such patterns are derived from the interaction between form and motion systems and in Experiment 2 we question whether this process is dependent on visual awareness. In Experiment 3 we conducted a control experiment to determine whether highly coherent patterns are simply detected faster by the visual system as their contrast is increased to break suppression.

## MATERIALS AND METHODS

### OBSERVERS

Six experienced observers participated in the present study. All had normal or corrected to normal visual acuity and were naïve to the aims of the present study.

#### Stimuli

Stimuli were either Glass or GDM patterns consisting of 250 white dots (radius: 0.056°, 60 cd/m^2^), contained within a circular region (radius: 2°), placed on a mid gray background (30 cd/m^2^). GDM patterns consisted of a number of movie frames in which dots were displaced across frames to generate motion. Note for this stimulus a central circular region with a radius of 0.25° was left blank. On the first movie frame, dots were assigned random positions in the stimulus area. On the second and subsequent frames, dots were displaced to another spatial location at a fixed step size of 0.2° per frame. Each movie frame was shown for 50 ms without an inter stimulus interval. These procedures resulted in a dot speed of 4°/s. A proportion of dots were displaced in a direction consistent with the global motion pattern (signal dots), while remaining dots moved in random directions (noise dots). Two types of complex motion structures were examined, radial and rotational. Radial motion was produced by displacing dots along radii from the center of the stimulus area (to simulate either expanding or contracting motion), while rotational motion was produced by assigning dots trajectories tangent to radii, which could be in a clockwise or anticlockwise direction. Note that signal and noise dots did not have limited lifetimes, but they were randomly assigned at the beginning of each movie frame transition to avoid tracking. Dots that left the stimulus area were replotted back into the stimulus area to a random location.

Glass patterns were generated using analogous procedures to those used to generate GDM stimuli, but consisted of a single frame and dots were paired to provide local orientation signals. To generate a Glass pattern, initially, 125 dots were randomly placed within the stimulus area and “partner dots” were assigned to them to generate a dot-pair or dipole; dots forming a dipole were separated by a fixed distance of 0.064°. Signal dipoles were assigned local orientations that were consistent with radial or rotational structure, while noise dipoles were randomly oriented.

These stimuli were generated using custom software written in MATLAB (version 2013b) on a Macintosh iMAC 2.8GHz computer. Stimuli were viewed on a linearized 23-inch “True3Di” 3D monitor (driven at a frame rate of 80 Hz) viewed through polarized lenses at a viewing distance of 57 cm.

#### Procedure

To suppress Glass or GDM patterns from visual awareness, they were presented to the right eye while a high-contrast (Weber contrast range of 0.5–1) and textured Mondrian pattern (see **Figure 1**) was presented to the left eye. This pattern was square in shape (subtending a visual angle of 8°) and consisted of colored rectangles of different sizes that randomly changed at a temporal rate of 10 Hz. The suppressed stimulus (right eye) was not presented in the center of the masked area, but from trial to trial was randomly assigned 1 of 4 locations which were either top-left, top-right, bottom-left, and bottom-right. This prevented observers from anticipating the location of the stimulus. We only considered break times for patterns whose position was correctly identified by the observer. If observers incorrectly indicated the position of the pattern, another trial was given to the observer. Both the stimulus and the Mondrian mask were bordered by thick black lines (6 cd/m^2^, width: 0.25°, length: 8.25°). The black lines aided the fusion of the images and minimized convergence.

As the goal of the present study was to investigate whether coherent global form and motion patterns are processed without visual awareness, we measured the time required for these patterns to break suppression (see Jiang et al., 2007). To achieve this on one trial either a Glass or GDM pattern was presented to observers under CFS suppression after a variable fore-period (that was randomized between 0.25 and 1 s) and the contrast of the pattern was gradually increased. This caused the pattern to break suppression. Initially the pattern contrast had a Weber contrast of 0.01 and this was increased at a rate of 0.02 per 100 ms. When the contrast reached 0.5, it remained at this value until the pattern broke suppression. The task of the observer was to press buttons corresponding to the locations of the stimulus when the pattern emerged into conscious vision such that it completely broke suppression. Throughout the trial the observer was instructed to maintain fixation on a white square presented at the center of the screen. The abovementioned procedures were repeated 10 times for both GDM and Glass patterns at 4 different pattern coherence levels of 0, 25, 50, and 100%, and for rotational and radial global patterns. Thus the experiment consisted of 80 trials that were performed in a randomized order.

## RESULTS AND DISCUSSION

**FIGURE 2:**
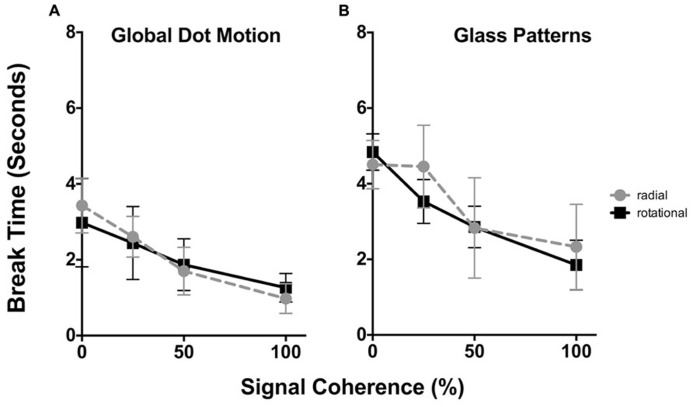
**The time taken (in seconds) for GDM (A) and static Glass patterns (B) to break CFS suppression plotted as a function of the signal coherence (%).** In each plot radial patterns are represented by circles, while rotational data is given by squares. Error bars signify 95% confidence intervals. For both GDM and static Glass patterns increasing signal coherence caused both patterns to break suppression faster. Note that overall break times for static Glass patterns were higher than GDM patterns.

The time taken for Static Glass and GDM patterns for radial and rotational structure to break suppression as a function of stimulus coherence is plotted in **Figures 2A,B** respectively. In each plot the average data for all six observers is shown. In each plot, radial structure are denoted by circles, while rotational structure as squares. Error bars signify 95% confidence intervals. A three-way repeated measures analysis of variance [ANOVA; comparing the effect of signal coherence, pattern type and structure type (4x2x2) on break times] reported main effects of both signal coherence [*F*(3,88) = 43.91, *p* < 0.0001] and structure type [i.e., form vs. motion, *F*(1,88) = 64.02, *p* < 0.0001], but not pattern type [i.e., radial and rotational, *F*(1,88) = 0.943), *p* = 0.334]. There were no significant interaction effects (ps > 0.481).

For both GDM and Static Glass patterns, a two-way repeated measures ANOVA was peformed to specifically examine whether signal coherence (factor 1, 0, 25, 50, and 100% coherence) and the global structure (factor 2, radial vs. rotational) affected break times. For GDM and static patterns there was a main effect of signal coherence [GDM: *F*(3,15) = 21.18, *p* < 0.0001; Static Glass patterns: *F*(3,15) = 39.95.75, *p* < 0.0001], but no effect of structure type [GDM: *F*(1,5) = 0.0255, *p* < 0.879; Static Glass patterns: *F*(1,5) = 0.711, *p* = 0.437]. There was no significant interaction indicating that the effect of signal coherence on break times was similar for both radial and rotational structures.

A number of findings present themselves. First, there was no difference in break times between rotational and radial patterns. This suggests that visual system does not prefer a particular global pattern, but rather treats them in a similar way when processing them under suppression. Second, increasing the signal coherence of the pattern significantly *reduced* break times for GDM patterns. As evident in **Figure 2A**, there is a monotonic decrease in break times as the coherence of the stimulus increases; break times decreased from approximately 3 to 1 s across the signal coherence range used in the present study. This replicates the findings of Kaunitz et al. (2013) who demonstrated that the global pooling of motion occurs without awareness such that highly coherent patterns broke suppression more often than random motion patterns as evidenced by higher d-prime values for 100% coherent patterns compared to 0% patterns. Our study differs from Kaunitz et al. (2013) as we focused on break times over a range of stimulus coherence levels. Third, we find a similar trend with the processing of static Glass patterns. When the stimulus coherence was increased (from 0 to 100% coherence) suppression break times decreased from approximately 4.5 to 2 s . These results are novel and demonstrate that visual system shows an advantage to highly coherent form patterns under CFS suppression. This finding implies that the visual system is able to detect and process the global form in Glass patterns without being inherently aware of the stimulus.

Evident in **Figure 2** and as indicated by the outcomes of the three-way ANOVA is that overall break times for GDM and Static Glass patterns are different; motion patterns break suppression faster than static patterns. As there was no difference in the results between radial and rotational patterns, they were combined and we reanalysed (two-way repeated measures ANOVA) our data to examine whether there was a significance difference in break times between GDM and static Glass patterns across the different levels of signal coherence. This analysis revealed that the break times between GDM and static Glass patterns were significantly different [*F*(1,88) = 64.02, *p* < 0.0001] and *post hoc* Holm–Sidak analyses (corrected for multiple comparisons at an alpha of 0.05) showed that GDM patterns broke suppression significantly faster (ps < 0.0034) across all signal coherence levels. This might suggest that motion information is detected faster than form patterns when suppressed from awareness under similar stimulus conditions. This observation might be accounted for by the fact that motion information (in particular fast speeds) has been shown to be fast-tracked (and bypassing V1) to motion centers in the visual cortex (in particular motion sensitive area, MT) for processing (see Ffytche et al., 1995). Thus, there is a processing advantage for motion over form under visually suppressed conditions.

Alternatively, faster break times for GDM stimuli (compared to static Glass patterns) might be because the temporal nature of motion might serve to attract attention which facillates the degree to which the pattern breaks suppression. This is possible given that the stimulus was presented in the periphery where the visual system is most sensitive to temporal change (e.g., McKee and Nakayama, 1984; Post, 1986) and might provide a means of drawing attention to these regions. To address this issue we conducted a supplementary experiment to examine the possibility that the dynamic nature of the motion stimulus might account for the differences in break times between static and motion patterns. We repeated Experiment 1, but with a “static-form” Glass pattern (at a signal coherence of 25%) that flickered on and off at a rate of 20 Hz (which was the same temporal rate of the motion stimulus). We compared CFS break times for this stimulus with both GDM and static Glass patterns as in the original experiment. If the dynamic nature of the GDM stimulus led to faster break times (by focussing attention), an expectation is that a flickering Glass pattern will similarly do so and break supression faster than were it static. Six observers participated in this supplementary experiment and break times were measured for GDM, static Glass patterns and flickering Glass patterns 16 times in a randomized order. Note that in this supplementary experiment observers were also tested with dynamic Glass patterns (see Experiment 2 for discussion).

**FIGURE 3:**
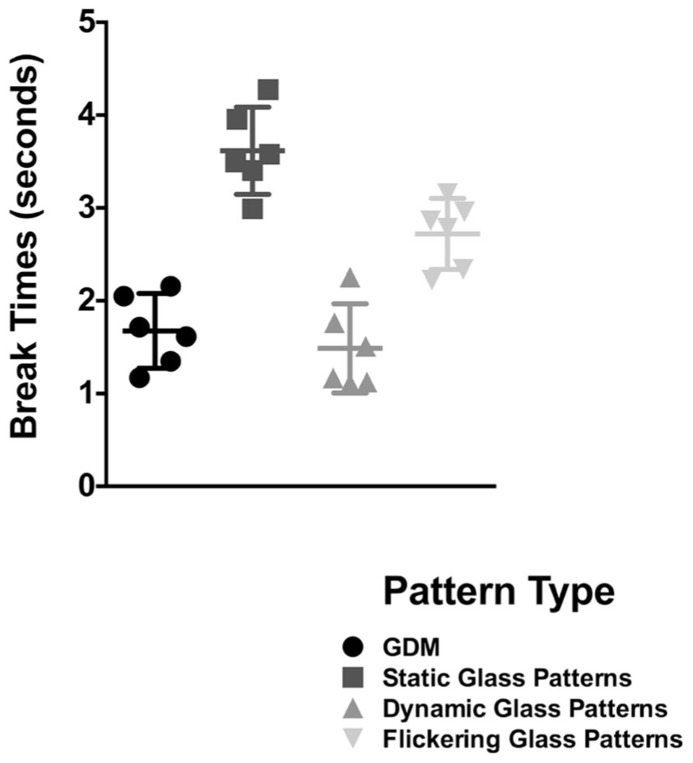
**Break times for GDM, static-, dynamic-, and flickering- Glass patterns as bar graphs.** Error bars represent 95% confidence intervals. Break times for a form-static flickering Glass patterns were lower than a continuous pattern, but were slower than GDM and dynamic Glass patterns which contain global motion.

In **Figure 3**, break times are plotted for the different stimulus types; error bars signify 95% confidence limits. Each symbol is representative of the break time for one observer for that condition. A repeated measures one-way ANOVA (with Sidak *post hoc* tests corrected for multiple comparisons between the different pattern types) revealed a significant effect of pattern type [*F*(3,15) = 45.01, *p* < 0.0001]. Replicating the findings of the original experiment (for a signal coherence of 25%), break times for GDM patterns were superior to that obtained with a static Glass pattern (*p* < 0.0001). However, while break times for a flickering Glass pattern was significantly faster than if it were static (*p* = 0.0303), it was significantly *slower* (*p* = 0.032) when compared to GDM stimuli. Thus, GDM patterns continue to be processed faster than static-form Glass patterns regardless of whether it was flickered at the same temporal rate. These findings suggest that while the dynamic change did reduce break times, it did not fully account for the difference in the processing of GDM and Glass pattern stimuli. This outcome might be indicative of a difference in the time course of processing form and motion information, and that motion patterns might be expediently processed and emerge into awareness from CFS suppression before static patterns.

In summary, the results of Experiment 1 demonstrate that the processing of static Glass patterns and GDM under visual suppression can occur without conscious awareness. Both global motion and form patterns break suppression faster when they are highly coherent. This suggests that the integration of local information in the processing of global form and motion information is automatic and occurs without the need to explicitly perceive or represent them consciously.

## EXPERIMENT 2: THE ROLE OF VISUAL AWARENESS IN THE PROCESSING OF DYNAMIC GLASS PATTERNS

In the previous experiment, we demonstrated that the processing of global form and motion can occur without visual awareness. Signal coherence was shown to significantly modulate break times. The implication here is that processing along both the dorsal and ventral visual pathways can function to process information without the need for the observer to be aware of the stimulus. Note that in Experiment 1, we necessarily considered the processing of motion and form separately. However, while it is convention to consider the functional processing of form and motion information as independent, recent studies have questioned this dichotomy. A large body of literature have provided strong evidence that, under appropriate stimulus conditions, the processing of form and motion are largely interactive (e.g., Lennie, 1980; Ross, 2004; Burr and Ross, 2006; Khuu, 2012; Khuu and Kim, 2013). A powerful demonstration of this effect is in the perception of *dynamic Glass patterns* (see Ross et al., 2000). Dynamic Glass patterns are constructed by presenting in rapid succession a series of independently generated Glass patterns conveying a particular form structure. As the position of dipoles in each pattern are randomly determined from frame to frame there is no net coherent motion, but there is a coherent form signal. Ross et al. (2000) demonstrated that while such patterns had no actual coherent motion, observers nevertheless perceived illusory global motion, though the global motion direction is ambiguous. They argued that the perception of this illusory global motion is an example of how “form can drive motion” and arises because the visual system treats the local orientation in the Glass patterns as “speed lines” or “motion streaks” which signals the axis but not the direction of motion (see Ross et al., 2000; Krekelberg et al., 2003; Or et al., 2007, 2010).

In Experiment 2 we examine whether the processing of dynamic Glass patterns occurs without visual awareness. As the processing of dynamic Glass patterns represents analysis and interaction between form and motion systems, examining the processing of this stimulus under CFS suppression provides a novel way of determining the relative contribution of visual awareness to this process. If the visual system required visual awareness for form and motion to interact in the processing of dynamic Glass patterns, then changing signal coherence might not modulate the degree to which it breaks suppression. This might be expected because the stimulus does not contain coherent motion. Alternatively if the processes through which form and motion interact (i.e., the treatment of dipoles as motion streaks) does not require visual awareness, we expect break times to decrease with signal coherence. This is likely given that we showed in Experiment 1 that the processing of static Glass patterns does not require visual awareness, and thus it may contribute to the processing of dynamic Glass patterns.

### METHODS

The same observers as in Experiment 1 participated in Experiment 2. Dynamic Glass patterns were generated using the abovementioned procedures used to generate Static Glass patterns. In particular at stimulus onset a Glass pattern was generated and displayed briefly to the observer for 50 ms, after which it was replaced with another randomly generated Glass pattern. This process continued until observers responded on the keyboard that it broke suppression. As in Experiment 1 the contrast of the pattern was initially increased and the trial lasted until the pattern broke suppression and became visible to the observer. As there was no difference in the type of global structure, only rotational patterns were tested and this pattern was presented at coherence levels of 0, 25, 50, and 100%. Each coherence level was tested 10 times in a randomized order.

## RESULTS AND DISCUSSION

**FIGURE 4:**
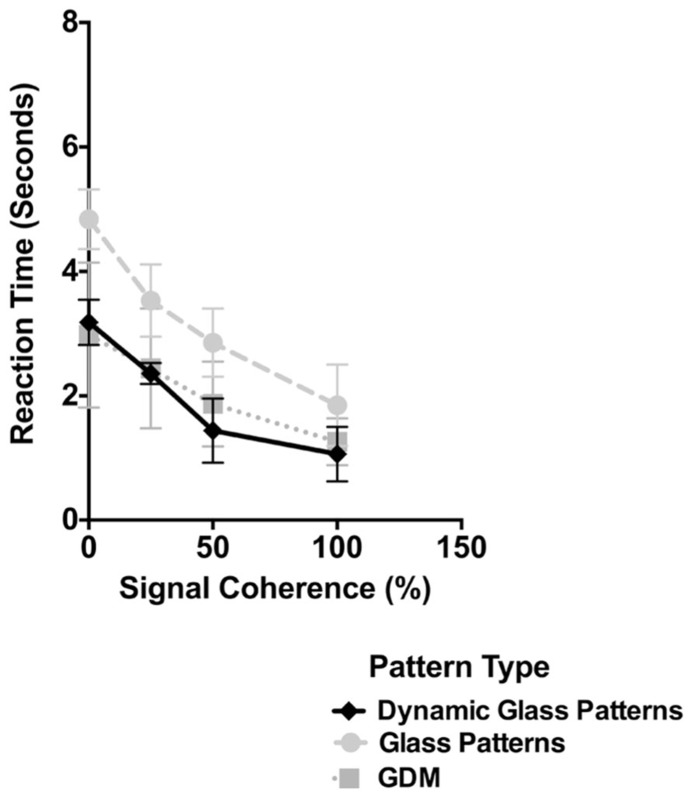
**The time taken for dynamic Glass patterns to break suppression (diamonds) is plotted as a function of the coherence of the stimulus.** Error bars represent 95% confidence intervals. Plotted for comparison are the data for GDM and Glass pattern stimuli from Experiment 1. Break times for dynamic Glass patterns decreased with signal coherence in a similar manner to those observed with GDM patterns.

The results of Experiment 2 are plotted in **Figure 4**. As in **Figure 2** the time taken for dynamic Glass patterns to break suppression (diamonds) is plotted as a function of the coherence of the pattern. Error bars represent 95% confidence intervals. For comparison the results from **Figure 2** for GDM and Static Glass concentric patterns are also included as light gray circles and squares respectively. As shown in **Figure 4** the time taken for dynamic Glass patterns shows a significant dependency on the coherence of the pattern (one-way repeated measures ANOVA, *F*(3,5) = 34.07, *p* < 0.0001). In particular break times significantly decreased as the signal coherence of the stimulus was increased from 0 to 100%; break times changed from approximately 3.5 to 1.5 s over this range. *Post hoc* Sidak’s multiple comparisons test which compared the mean break time for 0% coherence with the other coherence levels, indicated that, while there was no difference at 25% (*p* = 0.0511), the break times at 50 and 100% were significantly lower (ps < 0.0012). This data trend is consistent with those obtained with both GDM and Glass patterns and demonstrate, that there is an advantage to highly coherent patterns in the unconscious processing of dynamic Glass patterns. Thus, the results of Experiment 2 are consistent with the view that the interaction of form and motion can largely occur without visual awareness.

In **Figure 4**, the results for both GDM and Static Glass patterns are also plotted along with those obtained with dynamic Glass patterns. Evident in this figure is that the break times obtained with dynamic Glass patterns are similar to those obtained with GDM patterns and not with Static Glass patterns. This is despite the fact that dynamic Glass patterns do not contain coherent motion, but rather, like Static Glass patterns, consist of coherent form structure. A two-way repeated measures ANOVA comparing break up times for the three pattern types across the four different coherence levels revealed no significant interaction effects [*F*(6,40) = 1.21, *p* = 0.3209], but a main effect of signal coherence [*F*(3,20) = 60.06, *p* < 0.0001] and pattern type [*F*(2,40) = 28.45, *p* < 0.0001]. *Post hoc* Holm–Sidak’s multiple comparisons test showed a significant difference in break times between dynamic and Static Glass patterns (*p* < 0.0001), but not with GDM patterns (*p* = 0.4964). Though correlative, these data imply that the processing of dynamic Glass patterns might be similar to the processing of motion patterns, and most likely mediated by the same mechanism (see General Discussion), but with input from the form system.

Note that in the supplementary experiment that accompanied Experiment 1 (see **Figure 3**), break times were also measured for dynamic Glass patterns. Importantly, break times for this stimulus were significantly faster than flickering Glass patterns (*p* = 0.008), but were no different from GDM patterns (*p* = 0.987). This finding suggests that it is not the dynamic nature of this stimulus per se that leads to faster break times, but rather dynamic Glass patterns are likely to be processed by motion mechanisms sensitive to the illusory motion in these patterns.

## EXPERIMENT 3: PREFERENCE FOR HIGH COHERENCY, CONTRAST MODULATION, OR VISUAL AWARENESS?

Note that in Experiments 1 and 2, while the stimulus was suppressed from awareness using CFS, the contrast of the stimulus was systematically increased to break suppression. As noted previously break time observed under CFS might reflect inherent differences in the detection time between different patterns (e.g., see Jiang et al., 2007; Stein and Sterzer, 2011; Wang et al., 2012). It is possible that highly coherent patterns are detected faster (as they are processed by global form and motion detectors), and thus allowing them to break suppression before patterns of low coherence. This provides an alternative account for the findings of Experiments 1 and 2 that is not attributed to the unconscious processing.

**FIGURE 5:**
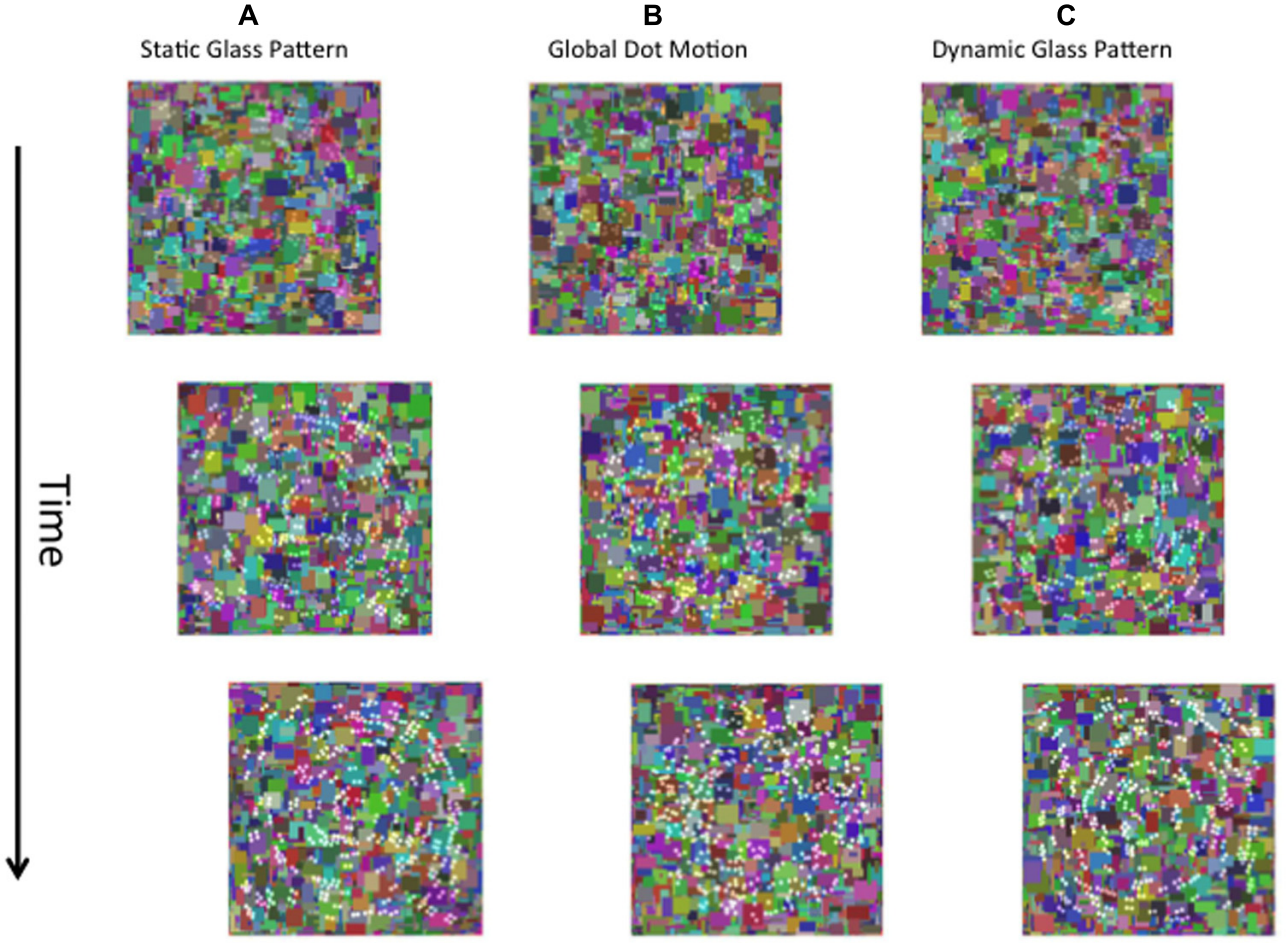
**A schematic diagram of the stimuli used in Experiment 3.** Contrast changing form and motion **(A–C)** stimuli were superimposed on dynamic Mondrian noise and presented to one eye.

To address this possibility we quantified the time taken for observers to detect patterns as they were made perceptible by changing contrast. The stimulus was the same as in Experiment 1 and 2, but the pattern was superimposed onto the dynamic CFS mask and presented monocularly to the left eye (see **Figure 5**). Observers were presented with GDM, static and dynamic Glass patterns at 0 and 100% coherent rotational structure. As in the previous experiments the contrast of the stimulus was systematically increased at a rate of 0.02 per 100 ms and observers had to respond as quickly as possible when the stimulus became visible. There were six stimulus conditions (three pattern types, and two coherence levels) which was repeated 10 times in a randomized order. Four of the six observers who participated previously acted as observers in this experiment.

## RESULTS

**FIGURE 6:**
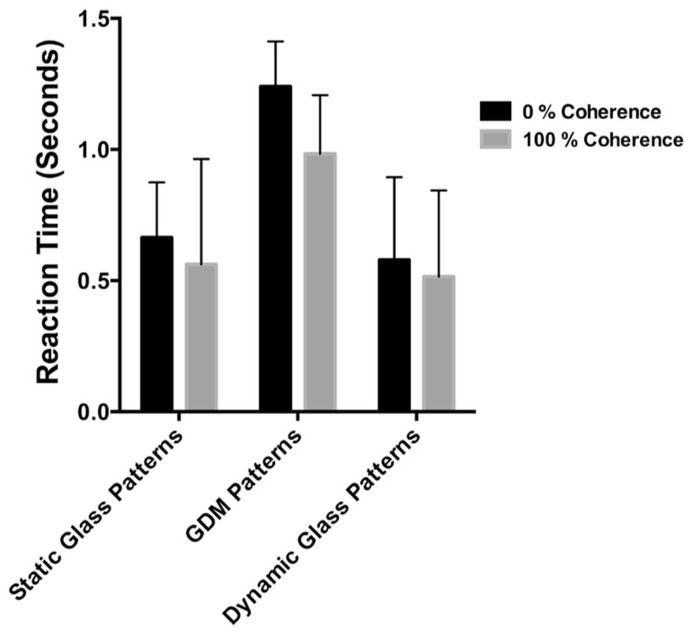
**The time taken to detect the stimulus from a dynamic background plotted for different pattern types.** Black bars represent conditions in which the pattern was 0% coherent, while gray bars the coherence level was 100%. Errors signify 95% confidence intervals. Detection times were different between pattern types, but not between highly coherent patterns compared to random patterns.

The results of Experiment 3 are shown in **Figure 6**. The average times required for the different pattern types to be detected by the observers are plotted as bar graphs for coherence levels of 0 and 100%. Error bars signify 95% confidence intervals. A two-way repeated measures ANOVA was performed on these data to determine whether there were differences in detection times between the pattern types and coherence levels. While the detection times for the three pattern types were significantly different [*F*(2,12) = 20.40, *p* = 0.0001], there was no significant difference between 0 and 100% coherence conditions [*F*(1,6) = 3.704, *p* = 0.1026). Note however, that 100% coherence patterns are consistently lower than 0% over for the three pattern types despite not reaching statistical significance. Previous studies have reported an advantage for highly coherent patterns when detecting them in noise (see Stein and Sterzer, 2011; Wang et al., 2012), but for our data they do not provide a complete account of the break times from suppression. Note that detection times overall are much faster, and the differences between 0 and 100% much smaller, than the break times reported in Experiment 1 and 2. Additionally, note that break times were significantly higher for static than for the two patterns containing dynamic information. *Post hoc* tests revealed that detection times for static Glass patterns were significantly different from GDM (*p* = 0.0006) and dynamic Glass patterns (*p* = 0.0002), but there was no difference between GDM and dynamic Glass patterns (*p* = 0.682). The similarities between GDM and dynamic Glass pattern perception, suggests that a common mechanism underlies their processing. In conclusion the findings of Experiment 3 indicated that the degree to which highly coherent patterns break CFS suppression is not due to a difference in contrast sensitivity, but rather is most likely attributable to the visual system processing global form and motion patterns outside of visual awareness.

## GENERAL DISCUSSION

In the present study we investigated the degree to which global form and motion patterns were processed without visual awareness. Using the CFS break paradigm and GDM and Glass patterns to selectively probe global form and motion processing, we provided evidence that their analysis can occur without visual awareness. In particular the degree to which these patterns broke suppression was shown to be dependent on their signal coherence. Highly coherent patterns broke suppression faster than patterns of low stimulus coherence. While both pattern types exhibited this data trend, global motion patterns broke suppression faster than static form patterns (flickering or stationary see Experiment 1). We additionally show in Experiment 2 that the interaction of form and motion can also occur without visual awareness. Using dynamic Glass patterns, in which the percept of motion coherence is driven by the form coherence of the pattern, we showed that changing the coherence of the pattern also modulated the degree to which it broke CFS suppression. Moreover, the degree to which dynamic Glass patterns broke suppression was similar to the detection of global motion rather than form.

Our findings are similar to those of Mudrik et al. (2011), who also provided evidence that form processing can occur without visual awareness. Using a similar suppression procedure to the present study they demonstrated that break times were much faster for incongruent (objects on an inconsistent background) than congruent patterns. This suggests that the processes critical to parsing the visual scene into meaningful objects and the background operates implicitly without the need to be driven by visual awareness. Our results agree with this since the processing of global form is an important step in the recognition of object shape. Our findings are consistent with those of Wang et al. (2012) who showed a detection advantage to Kanizsa figures configured so that local tokens are perceptually grouped to induce illusory contours. Here the visual system is able to integrate illusory contours without visual awareness which causes the stimulus to break faster than a null condition in which no illusory contours were present. Finally, Sweeny et al. (2011) demonstrated that the processing of contours arising from the grouping of a train of locally oriented elements, occurs without visual awareness. Our study is consistent with Sweeny et al. (2011) as the processing of contours and Glass patterns are integral steps in the recognition of object shape and together these studies demonstrate that the analysis of shape can occur without visual awareness.

As noted in the introduction, the analysis of faces largely requires visual awareness, though as noted by others aspects of the faces such as facial expression, they might be processed without awareness (see Jiang and He, 2006; Adams et al., 2010; Yang et al., 2010). As the detection of form is integral to the recognition of faces, our results are at first hand not in agreement with these findings. A possible account for this difference in findings is that the detection of global form in Glass patterns and faces occurs at different stages in visual processing. The processing of Glass patterns is likely to occur in areas V1 and V4 of the ventral route of processing (Gallant et al., 1996; Wilson et al., 2000), while faces are thought to be processed in higher cortical areas along the inferior temporal cortex such as the fusiform gyrus (Kanwisher et al., 1997; Liu et al., 2010). It could be argued that the processing of Glass patterns represents analysis at lower stages that automatically provide an indication of basic features such as orientation, shape and contours (see Sweeny et al., 2011), which then feeds into face processing areas. However, as complex meaning and recognition can be derived from faces and certain aspects of this process might dependent on visual awareness (Yang et al., 2010). However, future research would be fruitful in understanding this process.

The present study demonstrated that the interaction between form and motion can occur without awareness. It is believed that the perception of dynamic Glass patterns arises because the visual system treats local orientations in the Glass pattern as motion streaks which indicate the axis of motion, but not its direction (see Geisler, 1999). As demonstrated by the present study such motion streaks are integrated without the requirement of visual awareness and used by the motion system to derive an estimate of global motion. Additionally, the concordance between the processing of dynamic Glass patterns and GDM patterns agrees with this observation. This is further supported by previous studies that have demonstrated that dynamic Glass patterns can also directly influence by global motion. For example, Or et al. (2007); see also Nankoo et al. (2012) have shown that coherence thresholds (minimum signal required to perceive the global pattern) for dynamic Glass patterns are similar to GDM thresholds and not to static Glass patterns. Moreover, Burr and Ross (2006) has shown that dynamic Glass patterns interferes with the ability to detect global motion such that motion coherence thresholds increase with the addition of dynamic noise. Krekelberg et al. (2003) additionally have shown that dynamic Glass patterns selectively activate the same neural areas in the dorsal pathway that are responsible for the processing of motion. These studies and the present study suggests that a common system exists for the processing of dynamic Glass patterns and global motion.

Fang and He (2005) demonstrated that while the response of ventral regions of the visual cortex remain relatively attenuated to visually suppressed faces, dorsal areas remain active to invisible stimuli such as tools which have implied motion. In support, Almeida et al. (2010) demonstrated that the priming effects of images of tools continue to occur when the image is suppressed from awareness using CFS. However, recent studies have had difficulty replicating the findings of Almeida et al. (2010; e.g., Hesselmann and Malach, 2011), which might suggest that other stimulus factors might account for their findings. Yang and Blake (2012) have noted that the spatial frequency content of images is instrumental in modulating the degree to which the stimulus broke CFS suppression. In particular, stimuli consisting of low spatial frequency broke suppression faster and more often than high spatial frequencies. Both Fang and He (2005), Almeida et al. (2010) did not take into consideration the spatial frequency content of their stimulus, which might provide an alternative account for their findings. Indeed Sakuraba et al. (2012) have noted that low-level spatial features, such as stimulus length, modulated the degree to the stimulus broke CFS suppression. Thus, the conjecture that unconscious processing is exclusively limited to the dorsal pathway remains unresolved (see Mahon et al., 2013). The findings of Experiment 2 in the present study would argue that both pathways have the potential of processing information without visual awareness.

## Conflict of Interest Statement

The authors declare that the research was conducted in the absence of any commercial or financial relationships that could be construed as a potential conflict of interest.
